# *N*-{1,2-Bis(pyridin-3-yl)-2-[(*E*)-(pyridin-3-yl)methyl­idene­amino]­eth­yl}nicotinamide

**DOI:** 10.1107/S1600536813008544

**Published:** 2013-04-10

**Authors:** Claudia M. Quiroa-Montalván, Daniel Chávez, Reyna Reyes-Martínez, David Morales-Morales, Miguel Parra-Hake

**Affiliations:** a Centro de Graduados e Investigación del Instituto Tecnológico de Tijuana, Apdo. Postal 1166, 22500 Tijuana, BC, Mexico; b Instituto de Química, Universidad Nacional Autónoma de México, Circuito exterior, Ciudad Universitaria, México, DF, 04510, Mexico

## Abstract

In the title compound, C_24_H_20_N_6_O, the pyridin-3-yl groups on the ethyl­ene fragment are found in a *trans* conformation with a C(py)—C(e)—C(e)—C(py) (py = pyridine, e = ethylene) torsion angle of 179.2 (3)°. The dihedral angle between the pyridine rings is 3.5 (1)°. In the crystal, N—H⋯N and C—H⋯O=C inter­actions form a layer arrangement parallel to the *bc* plane. The compound displays disorder of the ethyl­ene fragment over two positions with an occupancy ratio of 0.676 (7) to 0.324 (7) that extends into the amide section of the nicotinamide moiety.

## Related literature

For supra­molecular structures, see: Nyburg & Wood (1964 ▶); House & Sadler (1973 ▶); Koçak (2000 ▶). For a related enanti­oselective catalyst, see: Jacobsen *et al.* (1990 ▶); Corey & Kühnle (1997 ▶); Corey *et al.* (1989 ▶). For coordination compounds with polypyridine ligands related to the title compound, see: Parra-Hake *et al.* (2000 ▶); Cruz Enríquez *et al.* (2012 ▶). For the synthesis of analogous compounds, see: Proskurnina *et al.* (2002 ▶); Tu *et al.* (2009 ▶); Irving & Parkins (1965 ▶).
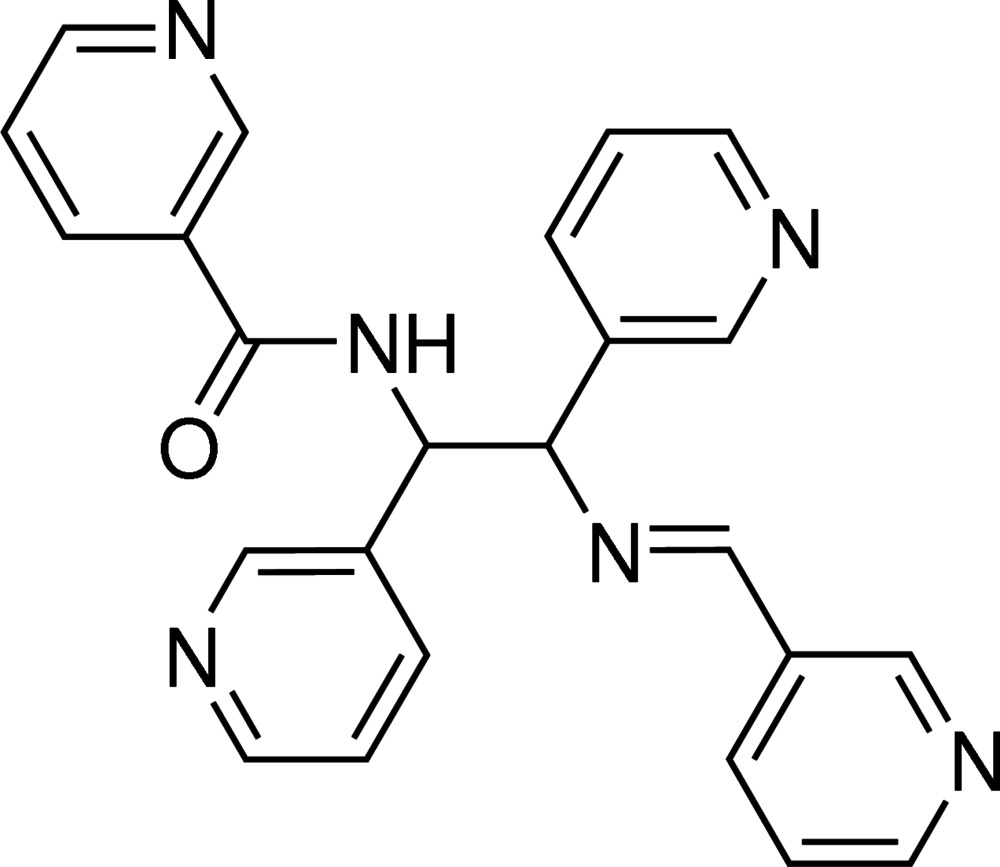


## Experimental

### 

#### Crystal data

C_24_H_20_N_6_O
*M* *_r_* = 408.46
Monoclinic, 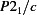

*a* = 11.4868 (17) Å
*b* = 8.7275 (13) Å
*c* = 21.105 (3) Å
β = 99.857 (3)°
*V* = 2084.6 (5) Å^3^
*Z* = 4
Mo *K*α radiation
μ = 0.08 mm^−1^
*T* = 298 K
0.28 × 0.26 × 0.14 mm

#### Data collection

Bruker SMART APEX CCD diffractometer
Absorption correction: multi-scan (*SADABS*; Bruker, 2007 ▶ *T*_min_ = 0.984, *T*_max_ = 0.992
17508 measured reflections
3821 independent reflections
2371 reflections with *I* > 2σ(*I*)
*R*_int_ = 0.050

#### Refinement

*R*[*F* ^2^ > 2σ(*F* ^2^)] = 0.071
*wR*(*F* ^2^) = 0.198
*S* = 1.02
3821 reflections
312 parameters
48 restraints
H atoms treated by a mixture of independent and constrained refinement
Δρ_max_ = 0.34 e Å^−3^
Δρ_min_ = −0.25 e Å^−3^

### 

Data collection: *SMART* (Bruker, 2007 ▶); cell refinement: *SAINT* (Bruker, 2007 ▶); data reduction: *SAINT*; program(s) used to solve structure: *SHELXS97* (Sheldrick, 2008 ▶); program(s) used to refine structure: *SHELXTL* (Sheldrick, 2008 ▶); molecular graphics: *ORTEP-3 for Windows* (Farrugia, 2012 ▶) and *DIAMOND* (Brandenburg, 2006 ▶); software used to prepare material for publication: *SHELXTL* and *PLATON* (Spek, 2009 ▶).

## Supplementary Material

Crystal structure: contains datablock(s) I, global. DOI: 10.1107/S1600536813008544/zl2533sup1.cif

Structure factors: contains datablock(s) I. DOI: 10.1107/S1600536813008544/zl2533Isup2.hkl

Supplementary material file. DOI: 10.1107/S1600536813008544/zl2533Isup3.cml

Additional supplementary materials:  crystallographic information; 3D view; checkCIF report

## Figures and Tables

**Table 1:** Hydrogen-bond geometry (Å, °)

*D*—H⋯*A*	*D*—H	H⋯*A*	*D*⋯*A*	*D*—H⋯*A*
N8—H8⋯N25^i^	0.84 (3)	2.33 (3)	3.168 (4)	174 (3)
C28—H28⋯O1^ii^	0.93	2.25	3.163 (16)	169

Symmetry codes: (i) 
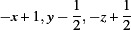
; (ii) 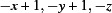.

**Fig. 1.**
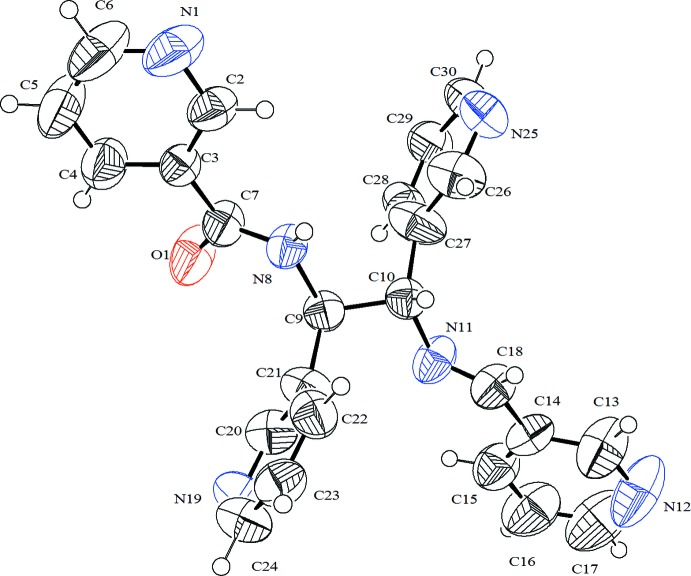
The molecular structure of the title compound I with displacement ellipsoids at the 50% probability. The minor fraction of the disorder was omitted.

**Fig. 2.**
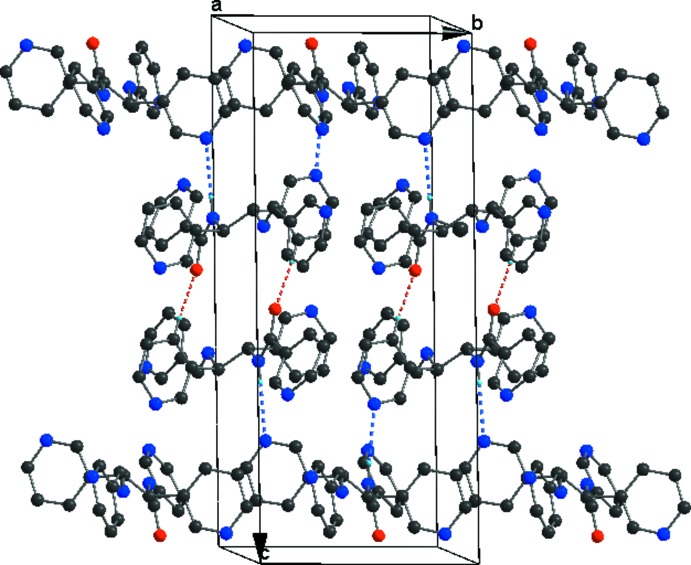
Representation of hydrogen bonds (N—H···N and C—H···O=C) found in the structure of the title compound. The hydrogen atoms not involved in the hydrogen bond interactions were omitted.

**Fig. 3.**
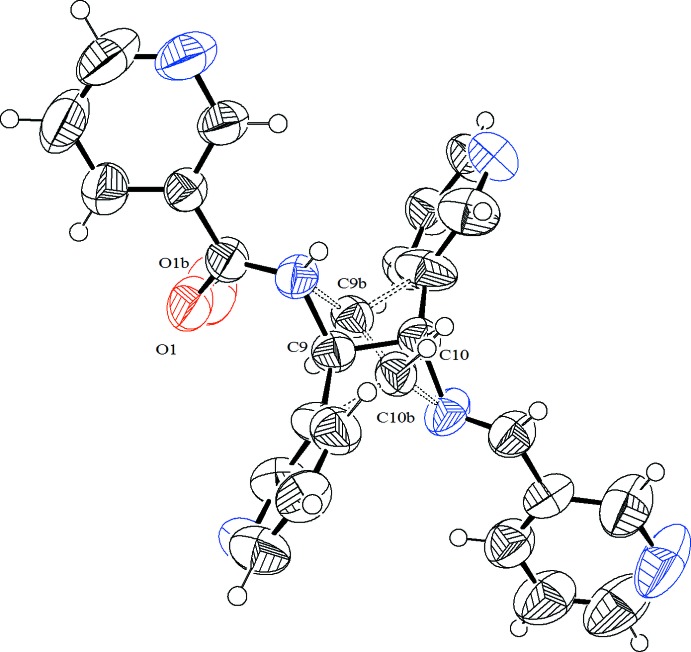
The major and minor component of the disorder of compound I, dashed lines indicate the minor fraction. The displacement ellipsoids are at the 50% probability.

## supplementary crystallographic information

### Comment

1,2-Diaryl-1,2-diaminoethanes have long been used as complexation agents for transition metal ions (House & Sadler, 1973; Koçak, 2000; Nyburg & Wood, 1964). These compounds are also important building blocks in the design of enantioselective catalysts (Corey & Kühnle, 1997; Corey *et al.*, 1989; Jacobsen *et al.*, 1990). The synthesis of diamines involves the reaction of aromatic aldehydes with ammonia to produce hydrobenzamides and amarine (2,4,5-triphenyl-2,5-dihydro-1*H*-imidazole).

For some time we have been interested in the coordination chemistry of polypyridine ligands which may have fluorescent properties, could act as sensors for transition metal ions, or could be used as building blocks for the construction of different coordination polymers. Some of the compounds that have been studied for this purposes are: *cis*-(±)-2,4,5-tri(2-pyridyl)imidazoline (Parra-Hake *et al.*, 2000), 2,4,6-tri(2-pyridyl)-1,3,5-triazinane, 2,4,5-tri(2-pyridyl)imidazole, *trans*-(±)-2,4,5-tri(4-pyridyl)imidazoline and 2,4,5-tri(4-pyridyl)imidazole (Cruz Enríquez *et al.*, 2012, Koçak, 2000).

As a part of our ongoing research on the chemistry of polypyridine ligands, in our attempts to synthesize the ligand *cis*-(±)-3-(2,5-di(pyridin-3-yl)-4,5-dihydro-1*H*-imidazol-4-yl)pyridine, we have been able to isolate the title compound, (*E*)—*N*-(1,2-di(pyridin-3-yl)-2-(pyridin-3-ylmethyleneamino)ethyl)nicotinamide (I). 1,2-diaryl-1,2-diaminoethane analogues to the title compound are obtained from the reactions of aromatic benzaldehydes with ammonia (Irving & Parkins, 1965; Proskurnina *et al.*, 2002; Tu *et al.*, 2009). The structure of the title compound I with the atom numbering is shown in Figure 1.

In the title compound I, C_24_H_20_N_6_O, the ethylene fragment presents a *trans* conformation between the two pyridin-3-yl groups with a torsion angle of 179.2 (3)° [C27—C10—C9—C21], and the nicotinamide groups presents a torsion angle of 175.1 (3)° [N1—C10—C9—N8].

Compound I has an imine group with a C—N distance of 1.329 (4) Å and the crystal structure is stabilized by hydrogen bonds (N—H···N and C—H···O=C). The hydrogen bond between the carboxyl group and the C—H bond produces a centrosymmetric dimer with a H···O distance of 2.25 Å. The dimers are further connected by N—H···N interactions between the imine group and one pyridine N-atom, and these interactions give rise to a layer arrangement parallel to the *bc* plane. The ethylene group (C9—C10) and the oxygen (O1) atom exhibit a statistical orientational disorder, Figure 3. The statistical fractions of the major and minor disordered components refined to 0.676 (7) and 0.324 (7) for the ethylene group (C9—C10), and 0.61 (6) and 0.39 (6) for the oxygen atom (O1).

### Experimental

The synthesis of the title compound included reagent grade starting materials and solvents. A mixture of 2 ml of pyridine-3-carboxaldehyde and 8.17 g of ammonium acetate was heated to 120 °C under stirring for 3 h. The reaction mixture was cooled and diluted with dichloromethane (50 ml), washed with water (3 *x* 30 ml) dried over MgSO_4_ and rotary evaporated, and crystallized by gas phase diffusion of diethyl ether into dichloromethane, providing yellow crystals. IR (KBr pellet) 3240, 3038, 3853, 1651, 1630, 1584, 1530, 1421, 1322, 1024, 804, 710 cm^-1^. ^1^H NMR (CDCl_3_-*d*_6_, 200 MHz) δ 8.87 (d, *J*= 2.2 Hz, 1H), 8.86 (d, *J*= 2.6 Hz, 1H), 8.69 (dd, *J*= 2.0, 1.4 Hz, 1H), 8.67 (dd, *J*= 1.8, 1.2 Hz, 1H), 8.63 (d, *J*= 2.2 Hz, 1H), 8.52–8.45 (m, 3H), 8.35 (s, 1H), 8.12 (ddd, *J*= 8.0, 2.2, 1.8 Hz, 1H), 8.02 (ddd, *J*= 8.0, 2.2, 1.8 Hz, 1H), 7.62 (ddd, *J*= 8.0, 2.2, 1.8 Hz, 1H), 7.56 (ddd, *J*= 8.0, 2.2, 1.8 Hz, 1H), 7.41–7.16 (m, 5H), 5.68 (dd, *J*= 8.0, 5.6 Hz, 1H), 5.10 (d, *J*= 5.6 Hz, 1H). ^13^C NMR (CDCl_3_-*d*_6_, 200 MHz) δ 165.2, 161.6, 152.6, 152.5, 150.4, 149.8, 149.4, 148.9, 147.8, 136.4, 135.3, 135.2, 134.9, 133.2, 130.8, 129.7, 123.9, 123.6, 123.6, 123.3, 97.0, 74.5, 57.9.

### Refinement

H atoms were included in calculated positions (C—H = 0.93 Å for aromatic H, C—H = 0.97 Å for methyn H), and refined using a riding model, with *U*_iso_(H) = 1.2 *U*_eq_ of the carrier atom. H atoms on N were located in a Fourier map and refined with *U*_iso_(H) = 1.2 *U*_eq_(N).

The disorder was modelled by splitting atoms with the highest prolate anisotropic displacement parameters (ADPs) into two components; the naming convention used involved appending a "B" suffix to the index number, such that O1, C9 and C10 became O1B, C9B and C10B. To ensure a sensible geometry for the disordered model, the bond distances and angles along the ethylene and carbonyl moieties were restrained to be similar (instructions SAME and SADI), and the ADPs of the disordered atoms were also restrained to be similar (instruction SIMU), with an s.u. value of 0.01 Å^2^. Subject to these conditions, the refined occupancies for the two major components were 0.676 (7) for the ethylene moiety and 0.61 (6) for the oxygen atom.

The positions and displacement parameters of the rest of the atoms are sufficiently well defined to allow for a refinement without any additional positional or similarity restraints.

### Crystal data

**Table:**

C_24_H_20_N_6_O	*F*(000) = 856
*M**_r_* = 408.46	*D*_x_ = 1.301 Mg m^−^^3^
Monoclinic, *P*2_1_/*c*	Mo *K*α radiation, λ = 0.71073 Å
Hall symbol: -P 2ybc	Cell parameters from 3500 reflections
*a* = 11.4868 (17) Å	θ = 2.4–23.6°
*b* = 8.7275 (13) Å	µ = 0.08 mm^−^^1^
*c* = 21.105 (3) Å	*T* = 298 K
β = 99.857 (3)°	Prism, colourless
*V* = 2084.6 (5) Å^3^	0.28 × 0.26 × 0.14 mm
*Z* = 4	

### Data collection

**Table:**

Bruker SMART APEX CCD diffractometer	3821 independent reflections
Radiation source: fine-focus sealed tube	2371 reflections with *I* > 2σ(*I*)
Graphite monochromator	*R*_int_ = 0.050
Detector resolution: 0.661 pixels mm^-1^	θ_max_ = 25.4°, θ_min_ = 1.8°
ω–scans	*h* = −13→13
Absorption correction: multi-scan (*SADABS*; Bruker, 2007	*k* = −10→10
*T*_min_ = 0.984, *T*_max_ = 0.992	*l* = −25→25
17508 measured reflections	

### Refinement

**Table:**

Refinement on *F*^2^	Primary atom site location: structure-invariant direct methods
Least-squares matrix: full	Secondary atom site location: difference Fourier map
*R*[*F*^2^ > 2σ(*F*^2^)] = 0.071	Hydrogen site location: inferred from neighbouring sites
*wR*(*F*^2^) = 0.198	H atoms treated by a mixture of independent and constrained refinement
*S* = 1.02	*w* = 1/[σ^2^(*F*_o_^2^) + (0.0806*P*)^2^ + 0.8743*P*] where *P* = (*F*_o_^2^ + 2*F*_c_^2^)/3
3821 reflections	(Δ/σ)_max_ < 0.001
312 parameters	Δρ_max_ = 0.34 e Å^−^^3^
48 restraints	Δρ_min_ = −0.25 e Å^−^^3^

### Special details

**Table:**

Geometry. All e.s.d.'s (except the e.s.d. in the dihedral angle between two l.s. planes) are estimated using the full covariance matrix. The cell e.s.d.'s are taken into account individually in the estimation of e.s.d.'s in distances, angles and torsion angles; correlations between e.s.d.'s in cell parameters are only used when they are defined by crystal symmetry. An approximate (isotropic) treatment of cell e.s.d.'s is used for estimating e.s.d.'s involving l.s. planes.
Refinement. Refinement of *F*^2^ against ALL reflections. The weighted *R*-factor *wR* and goodness of fit *S* are based on *F*^2^, conventional *R*-factors *R* are based on *F*, with *F* set to zero for negative *F*^2^. The threshold expression of *F*^2^ > σ(*F*^2^) is used only for calculating *R*-factors(gt) *etc*. and is not relevant to the choice of reflections for refinement. *R*-factors based on *F*^2^ are statistically about twice as large as those based on *F*, and *R*- factors based on ALL data will be even larger.

### Fractional atomic coordinates and isotropic or equivalent isotropic displacement parameters (Å^2^)

**Table:**

	*x*	*y*	*z*	*U*_iso_*/*U*_eq_	Occ. (<1)
O1	0.3921 (12)	0.342 (4)	0.0332 (5)	0.109 (5)	0.61 (6)
O1B	0.388 (2)	0.408 (5)	0.0340 (9)	0.121 (7)	0.39 (6)
N1	0.1399 (3)	0.3383 (5)	0.18544 (14)	0.1025 (11)	
C2	0.2412 (3)	0.3745 (4)	0.16651 (14)	0.0731 (9)	
H2	0.2921	0.4413	0.1921	0.088*	
C3	0.2751 (2)	0.3191 (3)	0.11162 (12)	0.0571 (7)	
C4	0.2000 (3)	0.2207 (4)	0.07388 (16)	0.0878 (10)	
H4	0.2195	0.1815	0.0361	0.105*	
C5	0.0947 (3)	0.1806 (5)	0.0931 (2)	0.1138 (14)	
H5	0.0424	0.1127	0.0691	0.137*	
C6	0.0703 (3)	0.2443 (6)	0.1484 (2)	0.1124 (15)	
H6	−0.0012	0.2188	0.1607	0.135*	
C7	0.3861 (3)	0.3661 (4)	0.08965 (13)	0.0665 (8)	
N8	0.4788 (2)	0.4040 (3)	0.13361 (11)	0.0601 (6)	
H8	0.479 (3)	0.397 (3)	0.1736 (15)	0.072*	
C9	0.5966 (4)	0.4277 (5)	0.1140 (2)	0.0568 (12)	0.676 (7)
H9	0.5824	0.4637	0.0694	0.068*	0.676 (7)
C10	0.6602 (4)	0.5549 (6)	0.1553 (2)	0.0574 (12)	0.676 (7)
H10	0.6793	0.5219	0.2003	0.069*	0.676 (7)
C9B	0.5658 (7)	0.5179 (11)	0.1168 (4)	0.057 (2)	0.324 (7)
H9B	0.5616	0.5235	0.0701	0.068*	0.324 (7)
C10B	0.6859 (6)	0.4612 (10)	0.1491 (4)	0.059 (2)	0.324 (7)
H10B	0.6920	0.4578	0.1960	0.071*	0.324 (7)
N11	0.7701 (2)	0.5794 (3)	0.12864 (11)	0.0712 (7)	
N12	1.1564 (3)	0.7923 (6)	0.1683 (2)	0.1422 (16)	
C13	1.0551 (4)	0.7403 (5)	0.18534 (19)	0.1078 (13)	
H13	1.0438	0.7562	0.2274	0.129*	
C14	0.9677 (3)	0.6652 (4)	0.14390 (16)	0.0712 (8)	
C15	0.9841 (3)	0.6467 (4)	0.08213 (18)	0.0862 (10)	
H15	0.9262	0.5988	0.0525	0.103*	
C16	1.0847 (4)	0.6976 (6)	0.0634 (2)	0.1156 (15)	
H16	1.0960	0.6850	0.0211	0.139*	
C17	1.1665 (4)	0.7655 (7)	0.1062 (3)	0.1348 (19)	
H17	1.2356	0.7970	0.0926	0.162*	
C18	0.8618 (3)	0.6101 (4)	0.16693 (14)	0.0697 (8)	
H18	0.8634	0.5979	0.2108	0.084*	
N19	0.7524 (3)	0.1072 (3)	0.04909 (13)	0.0848 (8)	
C20	0.7026 (3)	0.2381 (4)	0.05998 (15)	0.0755 (9)	
H20	0.6810	0.3032	0.0251	0.091*	
C21	0.6799 (3)	0.2862 (3)	0.11762 (17)	0.0752 (9)	
C22	0.7164 (3)	0.1917 (4)	0.16992 (16)	0.0744 (9)	
H22	0.7054	0.2208	0.2109	0.089*	
C23	0.7687 (3)	0.0550 (4)	0.16045 (16)	0.0746 (9)	
H23	0.7932	−0.0112	0.1947	0.090*	
C24	0.7842 (3)	0.0183 (4)	0.10042 (18)	0.0846 (10)	
H24	0.8195	−0.0754	0.0944	0.102*	
N25	0.4976 (3)	0.8743 (3)	0.21511 (13)	0.0795 (8)	
C26	0.5503 (3)	0.7463 (4)	0.20567 (17)	0.0863 (10)	
H26	0.5746	0.6851	0.2416	0.104*	
C27	0.5731 (3)	0.6934 (4)	0.14883 (18)	0.0860 (11)	
C28	0.5332 (3)	0.7813 (4)	0.09498 (16)	0.0772 (9)	
H28	0.5458	0.7502	0.0546	0.093*	
C29	0.4749 (3)	0.9150 (4)	0.10240 (15)	0.0778 (9)	
H29	0.4467	0.9769	0.0673	0.093*	
C30	0.4591 (3)	0.9551 (4)	0.16320 (17)	0.0842 (10)	
H30	0.4185	1.0454	0.1680	0.101*	

### Atomic displacement parameters (Å^2^)

**Table:**

	*U*^11^	*U*^22^	*U*^33^	*U*^12^	*U*^13^	*U*^23^
O1	0.089 (4)	0.189 (14)	0.055 (3)	−0.054 (6)	0.026 (3)	−0.040 (4)
O1B	0.144 (10)	0.172 (17)	0.049 (6)	−0.066 (10)	0.022 (5)	0.009 (7)
N1	0.0616 (17)	0.170 (3)	0.0795 (19)	−0.006 (2)	0.0214 (15)	0.001 (2)
C2	0.0564 (17)	0.103 (2)	0.0609 (18)	0.0009 (16)	0.0132 (14)	0.0014 (16)
C3	0.0552 (15)	0.0628 (17)	0.0527 (15)	−0.0015 (13)	0.0074 (12)	0.0027 (13)
C4	0.074 (2)	0.111 (3)	0.078 (2)	−0.016 (2)	0.0106 (17)	−0.018 (2)
C5	0.076 (3)	0.149 (4)	0.113 (3)	−0.046 (3)	0.004 (2)	−0.012 (3)
C6	0.062 (2)	0.179 (4)	0.097 (3)	−0.026 (3)	0.017 (2)	0.020 (3)
C7	0.0663 (18)	0.090 (2)	0.0447 (16)	−0.0137 (16)	0.0145 (14)	−0.0084 (15)
N8	0.0593 (14)	0.0737 (16)	0.0497 (13)	−0.0127 (12)	0.0164 (12)	0.0006 (12)
C9	0.060 (3)	0.061 (3)	0.052 (2)	−0.004 (2)	0.0180 (19)	0.001 (2)
C10	0.062 (3)	0.062 (3)	0.050 (2)	−0.002 (2)	0.0136 (18)	−0.001 (2)
C9B	0.065 (4)	0.056 (5)	0.051 (4)	−0.003 (4)	0.016 (3)	−0.001 (4)
C10B	0.062 (4)	0.063 (5)	0.053 (4)	−0.007 (4)	0.012 (3)	−0.004 (4)
N11	0.0568 (14)	0.0999 (19)	0.0586 (14)	−0.0186 (14)	0.0144 (12)	0.0066 (13)
N12	0.085 (2)	0.204 (4)	0.131 (3)	−0.063 (3)	−0.001 (2)	0.024 (3)
C13	0.089 (3)	0.149 (4)	0.083 (2)	−0.028 (3)	0.006 (2)	0.015 (2)
C14	0.0514 (16)	0.084 (2)	0.078 (2)	0.0023 (16)	0.0122 (15)	0.0171 (17)
C15	0.072 (2)	0.100 (3)	0.088 (2)	−0.0076 (19)	0.0172 (18)	0.002 (2)
C16	0.089 (3)	0.166 (4)	0.100 (3)	−0.010 (3)	0.038 (3)	0.018 (3)
C17	0.081 (3)	0.187 (5)	0.142 (4)	−0.027 (3)	0.036 (3)	0.050 (4)
C18	0.0616 (18)	0.087 (2)	0.0602 (18)	−0.0030 (16)	0.0107 (15)	0.0063 (16)
N19	0.108 (2)	0.0748 (18)	0.0763 (18)	0.0132 (16)	0.0290 (16)	−0.0048 (15)
C20	0.078 (2)	0.076 (2)	0.077 (2)	0.0063 (18)	0.0268 (17)	0.0182 (17)
C21	0.092 (2)	0.0555 (17)	0.093 (2)	0.0013 (16)	0.0553 (19)	0.0079 (17)
C22	0.082 (2)	0.073 (2)	0.075 (2)	−0.0111 (17)	0.0341 (17)	−0.0086 (17)
C23	0.0673 (19)	0.081 (2)	0.076 (2)	0.0040 (17)	0.0114 (16)	0.0153 (17)
C24	0.098 (3)	0.069 (2)	0.093 (3)	0.0172 (19)	0.033 (2)	0.0021 (19)
N25	0.101 (2)	0.0716 (18)	0.0682 (17)	0.0071 (16)	0.0199 (15)	−0.0082 (14)
C26	0.109 (3)	0.071 (2)	0.090 (2)	0.010 (2)	0.046 (2)	0.0196 (18)
C27	0.116 (3)	0.0551 (18)	0.107 (3)	0.0028 (18)	0.075 (2)	0.0085 (18)
C28	0.094 (2)	0.071 (2)	0.075 (2)	−0.0136 (18)	0.0402 (18)	−0.0158 (17)
C29	0.082 (2)	0.082 (2)	0.0651 (19)	0.0154 (18)	0.0010 (16)	−0.0035 (17)
C30	0.100 (3)	0.076 (2)	0.075 (2)	0.0219 (19)	0.0088 (19)	−0.0157 (18)

### Geometric parameters (Å, º)

**Table:**

O1—C7	1.223 (8)	N12—C17	1.357 (6)
O1B—C7	1.234 (12)	C13—C14	1.379 (5)
N1—C6	1.307 (5)	C13—H13	0.9300
N1—C2	1.332 (4)	C14—C15	1.359 (5)
C2—C3	1.371 (4)	C14—C18	1.465 (4)
C2—H2	0.9300	C15—C16	1.358 (5)
C3—C4	1.372 (4)	C15—H15	0.9300
C3—C7	1.487 (4)	C16—C17	1.326 (6)
C4—C5	1.385 (5)	C16—H16	0.9300
C4—H4	0.9300	C17—H17	0.9300
C5—C6	1.365 (6)	C18—H18	0.9300
C5—H5	0.9300	N19—C20	1.316 (4)
C6—H6	0.9300	N19—C24	1.332 (4)
C7—N8	1.329 (4)	C20—C21	1.353 (4)
N8—C9B	1.495 (9)	C20—H20	0.9300
N8—C9	1.495 (5)	C21—C22	1.384 (5)
N8—H8	0.84 (3)	C22—C23	1.366 (4)
C9—C10	1.520 (5)	C22—H22	0.9300
C9—C21	1.556 (6)	C23—C24	1.348 (4)
C9—H9	0.9800	C23—H23	0.9300
C10—N11	1.483 (4)	C24—H24	0.9300
C10—C27	1.561 (6)	N25—C26	1.302 (4)
C10—H10	0.9800	N25—C30	1.314 (4)
C9B—C10B	1.514 (8)	C26—C27	1.352 (4)
C9B—C27	1.671 (11)	C26—H26	0.9300
C9B—H9B	0.9800	C27—C28	1.382 (5)
C10B—N11	1.526 (7)	C28—C29	1.368 (4)
C10B—C21	1.662 (10)	C28—H28	0.9300
C10B—H10B	0.9800	C29—C30	1.372 (4)
N11—C18	1.242 (3)	C29—H29	0.9300
N12—C13	1.354 (5)	C30—H30	0.9300
			
C6—N1—C2	116.5 (3)	N12—C13—H13	118.1
N1—C2—C3	124.0 (3)	C14—C13—H13	118.1
N1—C2—H2	118.0	C15—C14—C13	117.3 (3)
C3—C2—H2	118.0	C15—C14—C18	122.6 (3)
C2—C3—C4	118.0 (3)	C13—C14—C18	120.1 (3)
C2—C3—C7	123.3 (3)	C16—C15—C14	120.4 (4)
C4—C3—C7	118.6 (3)	C16—C15—H15	119.8
C3—C4—C5	118.8 (3)	C14—C15—H15	119.8
C3—C4—H4	120.6	C17—C16—C15	119.2 (4)
C5—C4—H4	120.6	C17—C16—H16	120.4
C6—C5—C4	117.7 (4)	C15—C16—H16	120.4
C6—C5—H5	121.1	C16—C17—N12	124.5 (4)
C4—C5—H5	121.1	C16—C17—H17	117.7
N1—C6—C5	125.0 (3)	N12—C17—H17	117.7
N1—C6—H6	117.5	N11—C18—C14	121.0 (3)
C5—C6—H6	117.5	N11—C18—H18	119.5
O1—C7—N8	123.4 (7)	C14—C18—H18	119.5
O1B—C7—N8	116.5 (13)	C20—N19—C24	115.5 (3)
O1—C7—C3	116.8 (8)	N19—C20—C21	125.7 (3)
O1B—C7—C3	122.5 (12)	N19—C20—H20	117.2
N8—C7—C3	118.6 (2)	C21—C20—H20	117.2
C7—N8—C9B	119.2 (4)	C20—C21—C22	117.0 (3)
C7—N8—C9	119.7 (3)	C20—C21—C9	114.4 (3)
C7—N8—H8	123 (2)	C22—C21—C9	127.7 (3)
C9B—N8—H8	113 (2)	C20—C21—C10B	130.0 (4)
C9—N8—H8	116 (2)	C22—C21—C10B	104.0 (4)
N8—C9—C10	108.1 (3)	C23—C22—C21	119.0 (3)
N8—C9—C21	117.0 (3)	C23—C22—H22	120.5
C10—C9—C21	108.7 (4)	C21—C22—H22	120.5
N8—C9—H9	107.6	C24—C23—C22	118.5 (3)
C10—C9—H9	107.6	C24—C23—H23	120.7
C21—C9—H9	107.6	C22—C23—H23	120.7
N11—C10—C9	104.1 (3)	N19—C24—C23	124.3 (3)
N11—C10—C27	115.5 (3)	N19—C24—H24	117.8
C9—C10—C27	106.1 (4)	C23—C24—H24	117.8
N11—C10—H10	110.3	C26—N25—C30	115.6 (3)
C9—C10—H10	110.3	N25—C26—C27	126.5 (3)
C27—C10—H10	110.3	N25—C26—H26	116.8
N8—C9B—C10B	105.6 (6)	C27—C26—H26	116.8
N8—C9B—C27	120.2 (6)	C26—C27—C28	117.0 (3)
C10B—C9B—C27	98.0 (7)	C26—C27—C10	113.9 (3)
N8—C9B—H9B	110.7	C28—C27—C10	128.2 (3)
C10B—C9B—H9B	110.7	C26—C27—C9B	131.9 (4)
C27—C9B—H9B	110.7	C28—C27—C9B	100.9 (4)
C9B—C10B—N11	103.0 (6)	C10—C27—C9B	46.6 (3)
C9B—C10B—C21	98.5 (7)	C29—C28—C27	118.6 (3)
N11—C10B—C21	119.5 (6)	C29—C28—H28	120.7
C9B—C10B—H10B	111.5	C27—C28—H28	120.7
N11—C10B—H10B	111.5	C28—C29—C30	118.1 (3)
C21—C10B—H10B	111.5	C28—C29—H29	121.0
C18—N11—C10	117.8 (3)	C30—C29—H29	121.0
C18—N11—C10B	118.0 (4)	N25—C30—C29	124.3 (3)
C13—N12—C17	114.7 (4)	N25—C30—H30	117.9
N12—C13—C14	123.8 (4)	C29—C30—H30	117.9

### Hydrogen-bond geometry (Å, º)

**Table:**

*D*—H···*A*	*D*—H	H···*A*	*D*···*A*	*D*—H···*A*
N8—H8···N25^i^	0.84 (3)	2.33 (3)	3.168 (4)	174 (3)
C28—H28···O1^ii^	0.93	2.25	3.163 (16)	169

Symmetry codes: (i) −*x*+1, *y*−1/2, −*z*+1/2; (ii) −*x*+1, −*y*+1, −*z*.

## References

[bb1] Brandenburg, K. (2006). *DIAMOND* Crystal Impact GbR, Bonn, Germany.

[bb2] Bruker (2007). *SAINT*, *SMART* and *SADABS* Bruker AXS Inc., Madison, Wisconsin, USA.

[bb3] Corey, E. J., Imwinkelried, R., Pikul, S. & Xiang, Y. B. (1989). *J. Am. Chem. Soc.* **111**, 5493–5495.

[bb4] Corey, E. J. & Kühnle, F. (1997). *Tetrahedron Lett.* **38**, 8631–8634.

[bb5] Cruz Enríquez, A., Figueroa Pérez, M. G., Almaral Sánchez, J. L., Höpfl, H., Parra-Hake, M. & Campos-Gaxiola, J. J. (2012). *CrystEngComm*, **14**, 6146–6151.

[bb6] Farrugia, L. J. (2012). *J. Appl. Cryst.* **45**, 849–854.

[bb7] House, D. A. & Sadler, W. A. (1973). *J. Chem. Soc. Dalton Trans.* pp. 1937–1941.

[bb8] Irving, M. N. H. & Parkins, R. M. (1965). *J. Inorg. Nucl. Chem.* **27**, 270–271.

[bb9] Jacobsen, E. N., Zhang, W., Loebach, J. L. & Wilson, S. R. (1990). *J. Am. Chem. Soc.* **112**, 2801–2803.

[bb10] Koçak, M. (2000). *Transition Met. Chem.* **25**, 231–233.

[bb11] Nyburg, S. C. & Wood, J. S. (1964). *Inorg. Chem.* **3**, 468–476.

[bb12] Parra-Hake, M., Larter, M. L., Gantzel, P., Aguirre, G., Ortega, F., Somanathan, R. & Walsh, P. J. (2000). *Inorg. Chem.* **39**, 5400–5403. 10.1021/ic000182y 11154599

[bb13] Proskurnina, M. V., Lozinskaya, N. A., Tkachenko, S. E. & Zefirov, N. S. (2002). *Russ. J. Org. Chem.* **38**, 1149–1153.

[bb14] Sheldrick, G. M. (2008). *Acta Cryst.* A**64**, 112–122. 10.1107/S0108767307043930 18156677

[bb15] Spek, A. L. (2009). *Acta Cryst.* D**65**, 148–155. 10.1107/S090744490804362X PMC2631630 19171970

[bb16] Tu, S.-J., Ai, T., Jiang, B., Wang, X., Shi, F., Ballew, A. & Li, G. (2009). *J. Org. Chem.* **74**, 9486–9489. 10.1021/jo902204s 19938854

